# ID-RDRL: a deep reinforcement learning-based feature selection intrusion detection model

**DOI:** 10.1038/s41598-022-19366-3

**Published:** 2022-09-13

**Authors:** Kezhou Ren, Yifan Zeng, Zhiqin Cao, Yingchao Zhang

**Affiliations:** School of Systems Sciences and Engineering, Sun Yat-Sen University, Guangzhou, 510006 China

**Keywords:** Computer science, Information technology

## Abstract

Network assaults pose significant security concerns to network services; hence, new technical solutions must be used to enhance the efficacy of intrusion detection systems. Existing approaches pay insufficient attention to data preparation and inadequately identify unknown network threats. This paper presents a network intrusion detection model (ID-RDRL) based on RFE feature extraction and deep reinforcement learning. ID-RDRL filters the optimum subset of features using the RFE feature selection technique, feeds them into a neural network to extract feature information and then trains a classifier using DRL to recognize network intrusions. We utilized CSE-CIC-IDS2018 as a dataset and conducted tests to evaluate the model’s performance, which is comprised of a comprehensive collection of actual network traffic. The experimental results demonstrate that the proposed ID-RDRL model can select the optimal subset of features, remove approximately 80% of redundant features, and learn the selected features through DRL to enhance the IDS performance for network attack identification. In a complicated network environment, it has promising application potential in IDS.

## Introduction

The accurate transmission of network traffic and the dependable operation of network systems are essential to economic development and growth. Modern networks are getting increasingly information-dense and complicated, and new networks such as the Internet of Things and the Internet of Vehicles are emerging^1^. Simultaneously, network assaults are getting increasingly diversified and covert, and resolving this issue has become a serious challenge for network security. An intrusion detection system (IDS) is a researcher-proposed and utilized tool for monitoring, detecting, and resolving network security issues. IDS has a positive influence on network security and cannot be ignored^2–4^.

In 1987, Denning developed an early intrusion detection system based on audit data and statistical approaches^5^. Based on their detection methodologies, IDS may be roughly divided into two categories: misuse-based and anomaly-based. The former uses a database of identified harmful patterns as its own detection method and identifies network attacks by comparing the input traffic with a database of known network attacks, but it cannot identify unknown network attacks due to the emergence of new network attacks such as zero-day attacks and DDoS; The latter identifies unknown harmful traffic using machine learning models trained on the dataset’s characteristics and the related labels, however the vast amount of redundant features and class imbalance in the intrusion detection dataset likely to result in a high false alarm rate for the models.

Some researchers have concentrated on standard machine learning approaches, such as support vector machines (SVM), artificial neural networks (ANN), and decision trees (DT)^1,6,7^. Although these methods are fast, they cannot extract the deep information inside network data and cannot effectively identify new network attacks^8^. Deep learning (DL) is a recently developed machine learning technology that can learn the profound properties of the original data using multi-layer neural networks and identify network assaults through continuous iterative training^9,10^. However, it is ineffective at recognizing undiscovered network attacks. Recursive feature elimination (RFE) and other feature removal approaches are claimed to be capable of obtaining the most valuable portion of the original data and enhancing the efficacy of network attack identification while lowering computing effort^11–13^.

Reinforcement learning (RL) is a suggested approach for machine learning that enables robots to reason and make decisions like humans^14^. It models issues using the Markov decision process (MDP), is capable of learning by active exploration and interaction with the environment, and is helpful in unfamiliar and hostile contexts. In recent years, some studies have combined deep learning and reinforcement learning to create deep reinforcement learning (DRL), which is capable of solving many complex practical problems using neural networks to fit the MDP process and is applicable in IDS, where cyber-attacks are becoming increasingly complex^15–17^.

This paper proposes ID-RDRL, an intrusion detection method with feature selection based on deep reinforcement learning, as a solution to the current issues faced by intrusion detection systems (IDS), such as large computation and poor recognition of unknown network attacks. ID-RDRL is a method for detecting intrusions based on deep reinforcement learning. Using RFE and DT, we first pick the ideal feature subset that best captures the deep information of the original data set, and then we utilize the Mini-Batch module to generate the data to accommodate the DRL model. Then, we create an effective network intrusion detection model. We utilize the CSE-CIC-IDS2018 dataset to train and evaluate the performance of ID-RDRL, and the experimental results demonstrate that our proposed strategy can successfully pick the best feature subset of the original dataset and further enhance the model's performance.

The described ID-RDRL model for intrusion detection has several benefits over previous ML models. The advantages include the following: (1) the neural networks used to implement the model: policy, value function, and Q function, which enable the network to adapt to new networks accurately and quickly; (2) the generated neural network models are suitable for distributed high-performance computing environments (e.g., Tensorflow, Pytorch); (3) the parameters are significantly reduced compared to deep learning networks with substantially fewer parameters, thereby reducing the complexity of the model; and (4) used for unsupervised learning applications.

The following are the primary contributions of this paper:

1. We present an intrusion detection system (IDS) based on feature selection and reinforcement learning, which can effectively pick the best subset of features and enhance the performance of the IDS for network attack identification, in particular identification of unknown network attacks.
2. We filter the most valuable subset of features using RFE and DT classifier to eliminate approximately 80% of duplicated features. Simultaneously, we apply DRL to supervised IDS, recode the data using Mini-Batch, make the supervised dataset relevant to the DRL model, and extract the profound link between features to increase the accuracy and efficiency of supervised IDS.
3. We built a comprehensive simulation experiment using Python and tested the performance of the model using the CSE-CIC-IDS2018 dataset, achieving an accuracy of 96.2% and an F1-score of 94.9%, respectively. In addition, we compare the proposed model to other prevalent ML models.

This paper is structured as follows: The “Related Works” Section describes the work related to feature selection methods for intrusion detection systems. The Work description presents the ID-RDRL model, the CSE-CIC-IDS2018 dataset, and data preparation methods. The ’Results” Section evaluates the model's performance using the dataset, while comparing the results of other ML models, and the “Conclusion” Section presents the discussion and conclusions. The abbreviated words and their corresponding full names appear in Table 1 and are arranged in the order in which they appear in the text.

**Table 1:** List of abbreviations.

Abbreviation	Full form	Abbreviation	Full Form
IDS	Intrusion Detection System	DRL	Deep Reinforcement Learning
DDoS	Distributed Denial of Service	SVM	Support Vector Machine
CNN	Convolutional Neural Network	KNN	K-Nearest Neighbor
KDD99	KDD CUP 99 Dataset	RF	Random Forest
DARPA98	DARPA Intrusion Detection DataSet(1998)	JSMA algorithm	Jacobian Saliency Map Attacks algorithm
ML	Machine Learning	LSTM	Long Short Term Memory
RFE	Recursive Feature Elimination	DL	Deep Learning
DT	Decision Tree	ANN	Artificial Neural Network
IoT	Internet of Things	DoS	Denial of Service
IVN	In-vehicle Networking	DM	Data Mining
DQN	Deep Q-Network	DDQN	Double Deep Q-Network
PG	Policy Gradient	AC	Actor Critical
MLP	Multilayer Perceptron	MDP	Markov Decision Process
Conv-AE	Convolutional AutoEncoder Network	ROC	Receiving Operating Characteristics Curve
AUC	Area Under the ROC Curve	RL	Reinforcement Learning
GBM	Gradient Boosting Machine	–	–

Sorting according to the order of appearance in the text.

## Related works

This section includes the most illustrative contemporary IDS research and a broad discussion on machine learning in network security research, particularly recent research on reinforcement learning and RFE feature extraction in IDS.

In the era of big data, machine learning approaches have been widely implemented in intrusion detection systems (IDS), and part of the research has employed classic machine learning algorithms or their enhancements, such as SVM, K-means, KNN, RF, and so on^1,18–20^, and deep learning algorithms, such as ANN, CNN, LSTM, etc^21–27^. In the literature^28^, the authors suggest an IDS based on spark and Conv-AE that employs public datasets such as KDD99 for performance evaluation, and the findings indicate that imbalanced datasets affect model performance. Ali et al.^29^ offer a novel intrusion detection system (IDS) based on fast learning networks (FLN) and the harmonic search algorithm (HSO) for IDS optimization, claiming that the IDS provides efficient and quick intrusion detection. Qureshi et al. proposed a novel adversarial intrusion detection system based on random neural networks (RNN-ADV). However, the perturbation environment significantly affects the performance of this model, which performs better in terms of accuracy and F1-score compared to deep neural networks when using the JSMA algorithm^30^. In the literature^31^, Safa et al. proposed a joint reinforcement learning-based intrusion detection system (FRL-IDS) for Internet of Things (IoT) networks in healthcare infrastructure. Their results demonstrated that the proposed model outperformed SVM-based IDS and was capable of identifying unknown network attacks.

Akhtar provided a CNN-based DoS intrusion detection model that got good results in DoS using the NSL-KDD dataset, but could only detect DoS network assaults and not unknown intrusions^9^. Mehedi et al.^10^ suggested an IDS model based on deep transfer learning with IVN, claiming the IDS was equivalent to a number of other current models. Compared to several other current models, its performance is superior. Fernando^32^ proposed a class rebalancing strategy based on a class balancing dynamic weighted loss function for the problem of uneven distribution of network attacks, claiming that experiments conducted using this method on highly unbalanced data demonstrated robust generalization, but the method did not include machine learning.

The CSE-CIC-IDS dataset family, proposed by the Canadian Cyber Security Laboratory^33^, has been extensively utilized in recent IDS research and is a family of intrusion detection datasets encompassing new forms of cyber threats. Thakkar et al. enumerate the many IDS datasets used to test IDS models, define the ML and DM approaches employed by IDS, and focus on two datasets, CIC-IDS-2017 and CSE-CIC-IDS-2018^2^, and study the performance of certain research on this dataset. It is difficult to visually compare the efficacy of individual research works on the dataset at IDS due to the fact that different classification criteria and validation methods were used. However, it has been determined that accuracy rates of 92% (multiclassification) and 94% (binary-classification) are the most desirable to date. In CIC-IDS2017^16^, Kamalakanta Sethi et al. introduced a novel IDS based on Deep Reinforcement Learning for IDS by merging Deep Q-Network and attention mechanism to detect and identify unidentified cyber assaults.

Some researchers have focused on the feature selection of the dataset, stating that preprocessing procedures, such as the feature selection of the data, are essential for the efficiency and performance of model training. Ons Aouedi et al. stated that determining the most significant characteristics to define network traffic is vital and conducted an in-depth analysis utilizing decision trees and feature selection techniques^34^. Wan et al. developed a robust fuzzy rough approximation space-based feature grouping and selection strategy utilizing graph theory (FGS-RFRAS), which was evaluated on 21 datasets to demonstrate that the method may enhance the model’s robustness^12^. Methods for feature selection may be categorized into three groups: filtering, embedding, and wrapper. Comparatively to the aforementioned feature selection methods, the wrapper method RFE may iteratively choose feature subsets and is better appropriate for IDS datasets with a huge data volume and numerous features. Yin et al.^11^ introduced IGRF-RFE for intrusion detection, which is regarded as a feature reduction strategy based on the filter feature selection method and packed feature selection method, and half of the features are filtered out by RFE while the multi-classification accuracy increases by 2%. Ripon presented a random forest and support vector machine (SVM) in combination with recursive feature elimination (RFE) to choose features for IDS, and the model was assessed using the NSL-KDD dataset^35^.

Reinforcement learning emphasizes the model's capacity to investigate the problem and is frequently implemented within decision models; IDS has been the subject of extensive research. Shi Dong et al. presented an optimization technique for network anomaly detection based on semi-supervised double-depth Q networks (SSDDQN), employing NSL-KDD and AWID datasets for training and attaining excellent results^19^. Lopez-Martin modified the classical DRL paradigm^36^ (based on the interaction with the environment) by replacing the environment with a sampling function of the recorded training invasion and applied it to the NSL-KDD and AWID datasets, as well as to the Deep Q Network (DQN), Double Deep Q Network (DDQN), the Policy Gradient (PG) and Actor-Critical (AC) were experimentally compared, and the experimental results indicated that the DDQN algorithm achieved the best results^37^. Meanwhile, Scott Emmons et al. researched offline reinforcement learning using supervised learning (RvS) techniques^17^. Their opinion that the optimal goal and reward settings are crucial to DRL success inspired us to set the reward to 0 or 1 in our simulation tests.

## Work description

**Figure 1:**
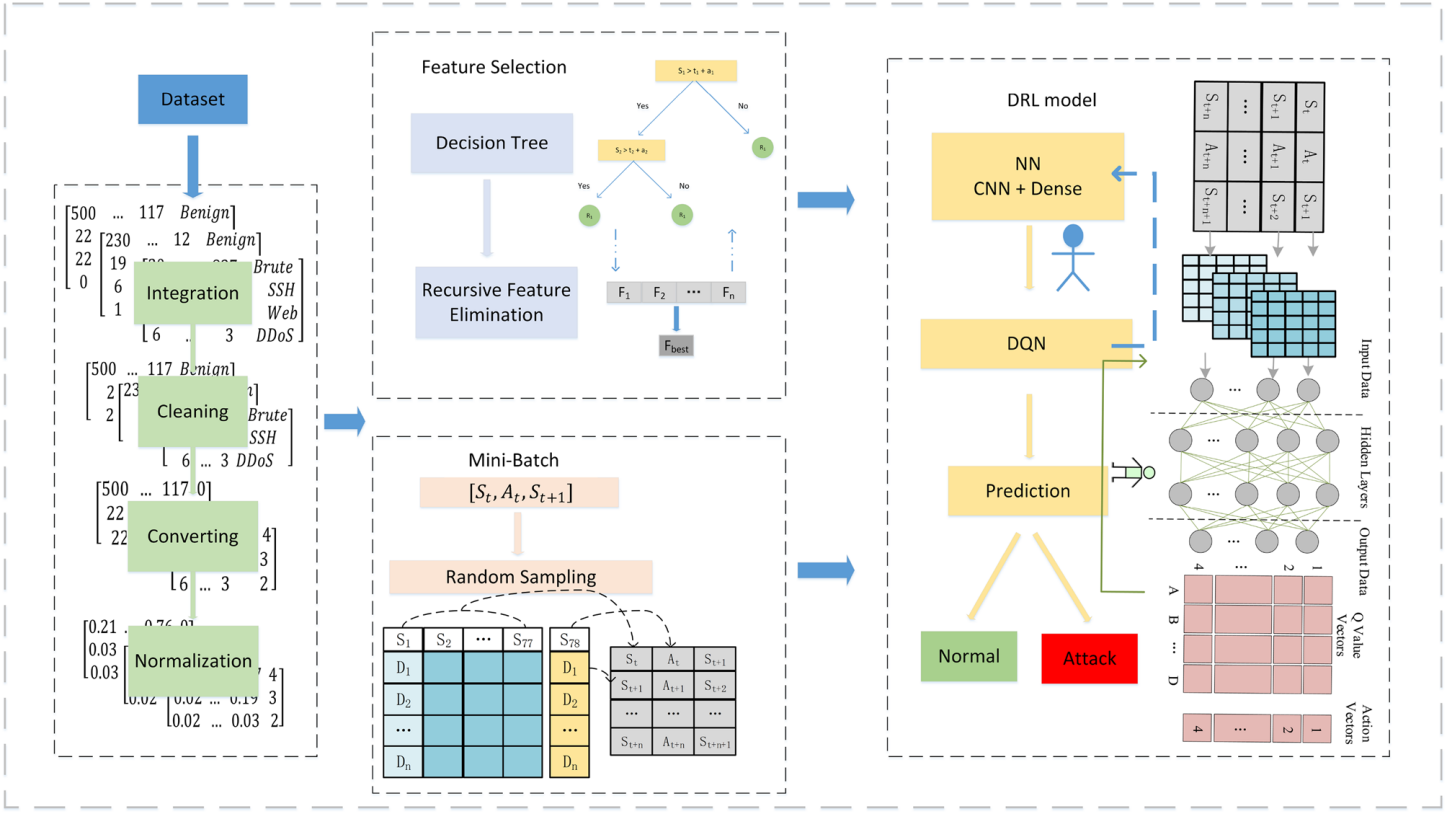
ID-RDRL Model schematic.

This section describes the many components of the entire effort, including the datasets and model components. Specifically, CSE-CIC-IDS2018 data is shown in the Intrusion detection dataset. At the same time, the suggested algorithm and framework are detailed in-depth in the Model description. Figure 1 depicts the overall architecture of the proposed reinforcement learning-based feature selection intrusion detection model (ID-RDRL). The strategy consists of two major components: feature selection and deep reinforcement learning. First, we preprocess the dataset and then enter the preprocessed data into the feature selection section to determine the optimal subset of features using DT + RFE. Next, in the Mini-Batch portion, the dataset is recoded by deleting redundant features based on the best feature subset, and the recoded data is put into the DRL model. Using reinforcement learning, the classifier is taught to categorize the traffic. The implementation of the technique is as detailed below.

1. The dataset is initially preprocessed, which consists of data integration, cleaning, transformation, and standardization. The processed data are transferred to the Feature selection stage in order to determine the ideal subset of features.
2. Using RFE and DT as classifiers, the input data are analyzed, sorted according to the relevance of the features, and the optimal subset of features is chosen.
3. The subset of selected characteristics is retained in the dataset, while redundant features are removed. Simultaneously, the data set is recoded based on Mini-Batch, and the recoded data samples are fed into the DRL model.
4. The data input to the DRL model will be retrieved by the neural network with feature information, followed by the DQN training of the classifier and the model predicting whether the input traffic is normal traffic or attack traffic.

### Intrusion detection dataset

**Figure 2:**
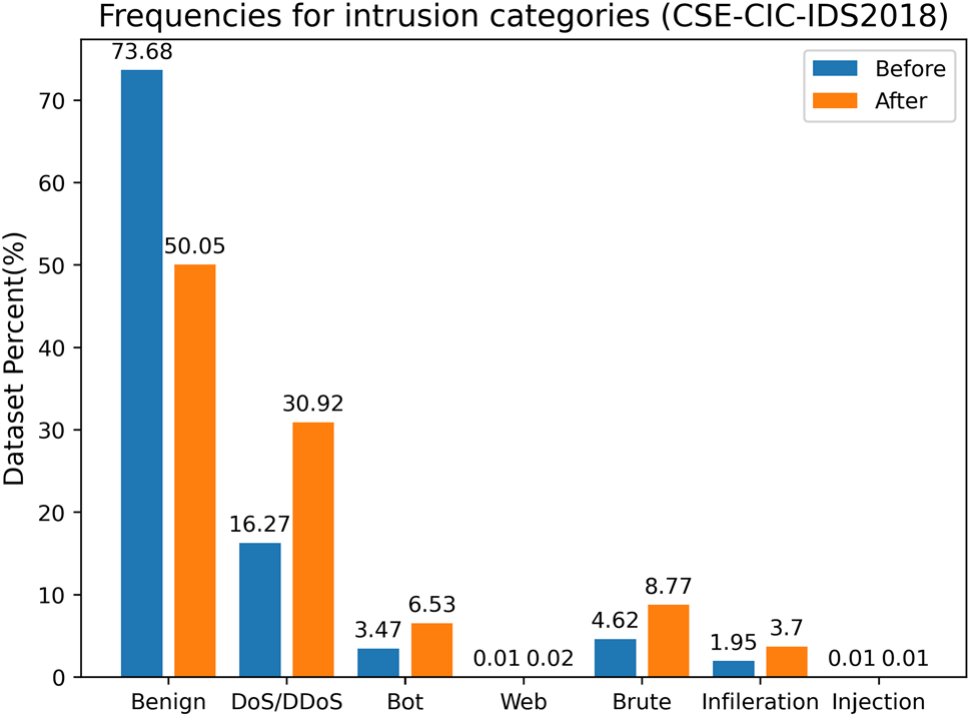
Frequencies for intrusion categories.

CSE-CIC-IDS2018 is one of the most recent IDS datasets. It includes seven distinct attack scenarios, including Brute-force, Heartbleed, Botnet, DoS, DDoS, Web assaults, and network penetration from within. The attacking infrastructure consists of 50 machines, whereas the infrastructure of the victim firm consists of 420 machines and 30 servers across five departments. The dataset contains each computer’s network traffic and system logs, as well as eighty characteristics collected from the recorded network traffic using CICFlowMeter-V3. Figure 2 depicts the proportion and distribution of each traffic category. The CSE-CIC-IDS2018 dataset has an unequal distribution of positive and negative samples, which is brought near to 50% by under-sampling Benign; the proportion of all classes before and after sampling is depicted in blue and orange, respectively, in Fig. 2. Since the dataset has 80 features, only a subset of them are displayed in Table 2, where the first column contains the feature's name and the second column contains a brief explanation.

**Table 2:** A part of features in the dataset.

Feature name	Feature short description
Dst Port	Destination port of connection
Protocol	Protocol used during connection
Timestamp	Time that connection occured
Flow duration	Duration that connection occurred
Tot Fwd Pkts	Total number of forward packets
Tot Bwd Pkts	Total number of backward packets
TotLen Fwd Pkts	Total length of forward packets
Fwd Pkt Len Max	Maximum length of forward packets
Bwd Pkt Len Mean	Mean size of packet in backward direction
Flow IAT Std	Standard deviation time between two packets sent in the forward direction
Fwd Seg Size Min	Minimum segment size observed in the forward direction
…	…
Active mean	Mean time a flow was active before becoming idle
Idle Std	Standard deviation time a flow was idle before becoming active
Idle Min	Minimum time a flow was idle before becoming active
Label	Describes if file is Attack or Benign

The first column is the name of the feature. The second column is the description corresponding to the feature.

### Model description

This section describes the feature selection RFE method and the DRL model investigated in this study. DRL is widely reported in the literature^14^. First, the CSE-CIC-IDS2018 dataset is preprocessed with data, then the optimal feature subset of the dataset is extracted using the RFE feature selection method combined with DT algorithm, the data is encoded and processed by the Mini-Batch module, and the encoded and processed data is input to CNN for additional feature extraction, and the DRL for final feature extraction. The processed data is fed into CNN for additional feature extraction, the training of the classifier enables the model to recognize network threats using DRL, and the performance of this IDS is then assessed.

### Dataset preparation

#### Data integration

The CSE-CIC-IDS2018 dataset is a raw data file comprised of 10 days of traffic collected from ten genuine networks. It contains 15 network assaults, including Slowloris DoS, SQL injection, and novel network attacks such as SSH Brute Force and DDoS. We combine the ten raw files to create a file containing 16,233,002 traffic samples for later training. As demonstrated in Fig. 2, by undersampling the dataset so that the normal: attack ratio is 1:1, the dataset comprises around 8,876,032 samples.

#### Data maintenance

Due to the fact that the samples in the original data set contain either missing values or duplicate values, about 2000 invalid samples were eliminated. Following data cleansing, a dataset with 77 columns of characteristics and 8,874,005 samples was produced.

#### Data transformation

Based on Fitni’s work^15^, we translated the 15 traffic attack categories in the original file into 7 types, including Benign, BruteForce, DoS, Bot, DDoS, Web Attacks, and Infiltration, as depicted in Fig. 2 for the 7 types of network traffic, where Benign is normal traffic.

#### Data normalization

Since some of the characteristics have a vast range of values and fluctuate dramatically from one feature to the next, e.g., “Port Number” runs from 1 to 65,535, while “Packet Size” goes from 1 to 5000, this impacts the model's performance and necessitates additional computational power. This impacts the performance of the model and necessitates more mathematical work. Using the normalizing procedure, we transform all original characteristics to 0 or 1 values.

1 $$ x^{{\prime }} = \frac{{x - x_{Min} }}{{x_{Max} - x_{Min} }} $$

where $x^{{\prime }}$ represents the normalized eigenvalue, $x$ represents the initial eigenvalue, $x_{Min}$ represents the minimal eigenvalue, and $x_{Max}$ represents the maximum eigenvalue.

#### Feature selection method

There is frequently more than one type of feature in a dataset, and the combination of these features can represent the essence of the data. However, selecting these features and removing unnecessary and redundant features that do not affect the model's performance is often crucial to improving the model's performance and ensuring its efficient operation.

Feature selection is a method of data dimensionality reduction that can increase the accuracy of machine learning (ML) models by identifying a subset of features that really contribute to the sample to represent the sample, as well as minimize the training time and computing cost of the model. Additionally, it should be emphasized that feature selection is distinct from feature extraction. The primary distinction is that the former (e.g., RFE algorithm) attempts to discover the best subset of features from the original feature set, whereas the latter removes the features and produces a new set of features (e.g., CNN extracted features).
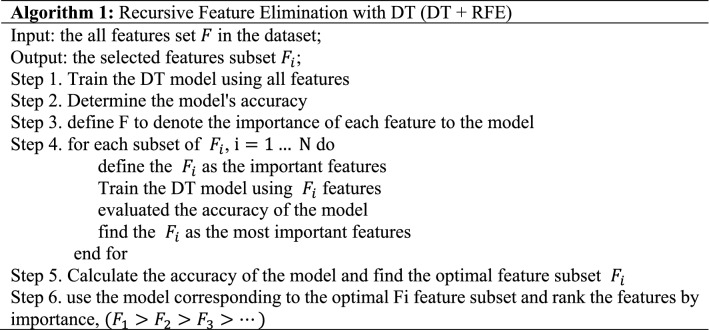


In this study, we begin by de-fitting the DT to the dataset, continually repeat the DT using the RFE method, and then rank each feature according to its relevance. Algorithm 1 describes the process of RFE to select the optimal feature subset in DT model by defining $F_{i}$ as the optimal feature subset $(F_{1} > F_{2} > F_{3} > \ldots )$, retaining the top-ranked $F_{i}$ feature subset each time, repeatedly fitting the model and evaluating the model's accuracy, and locating the $F_{i}$ feature subset with the optimal accuracy to be applied to the subsequent model as the feature selection result.

### Model detail

This section describes the process of Mini-Batching the feature-selected dataset and feeding it into DRL, where the data will first be fed into a CNN + MLP model for feature extraction, followed by training the model using reinforcement learning to enhance the performance of the IDS. Then the identification of network attacks will be completed.

#### Mini-Batch

DRL is often employed for unsupervised learning, however, the CSE-CIC-IDS2018 dataset is a supervised dataset with labels. To imitate the process of DRL, we attempt to treat all characteristics outside labels as states and labels as actions. Batch samples consisting of (1) feature states $S_{t}$, (2) label actions $A_{t}$, and (3) $S_{t + 1}$ are used in the training procedure. It should also be mentioned that the Mini-Batch Dataset is a subset of randomly selected samples from the dataset that are used as input data for the training model, and that the Mini-Batch Dataset is updated each time it is trained by randomly picking samples from the dataset.

**Figure 3:**
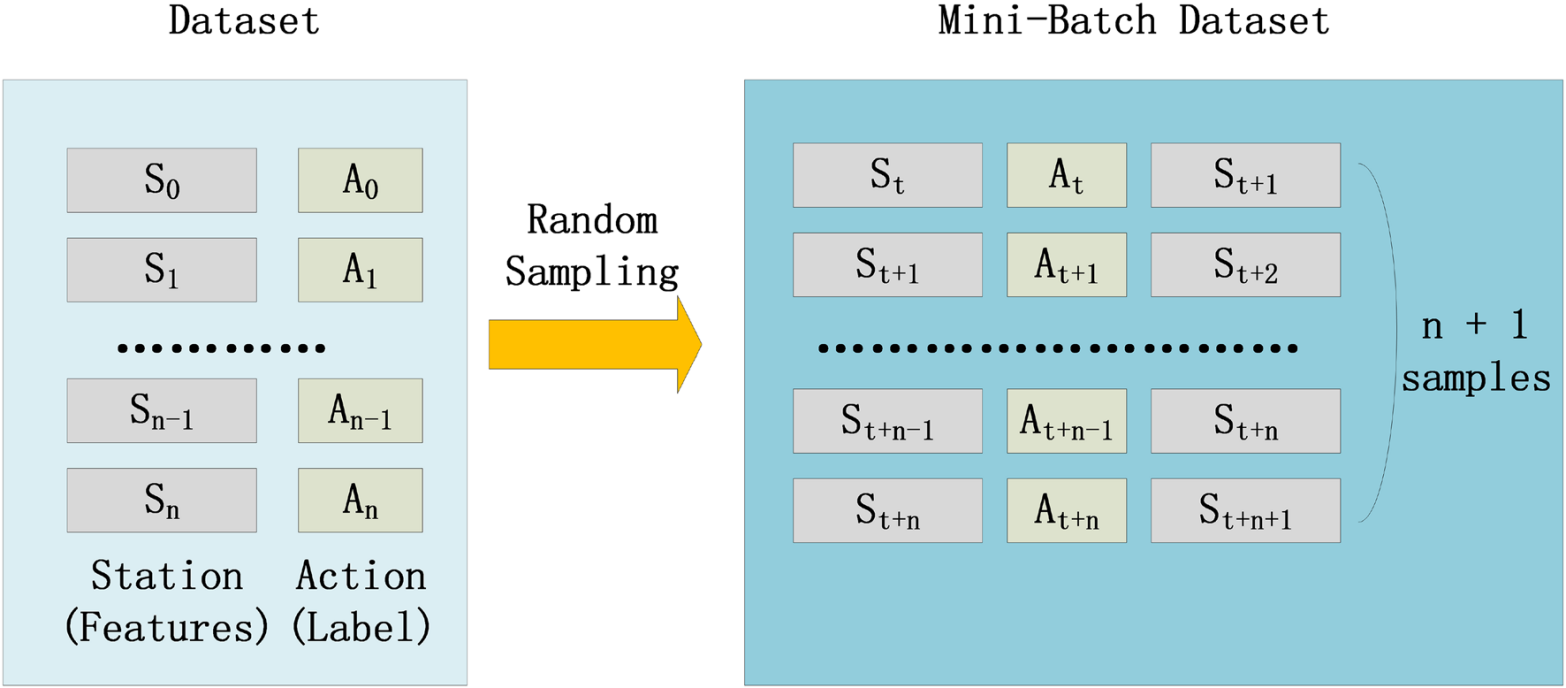
Mini-Batch data encoding schematic for DQN model.

Figure 3 depicts the structure of the Mini-Batch employed by ID-RDRL, which consists of $S_{t} , A_{t} , S_{t + 1}$ as the fundamental input data, with each batch is consisting of $n + 1$ instances of the structure described above. Each Mini-Batch consists of $n + 1$ consecutive samples chosen by indexing $t$, while the dataset is randomly disturbed before each training.

#### DQN model

Reinforcement learning is a machine learning technique based on the Markov decision process (MDP), which is a function consisting of S, A, T, and R, where S is a set of states, A is a set of actions, T is a mapping function for each state-action pair to transition to a new state, and R is the reward function obtained from this process. In the MDP, the transition from the current state-action pair to the next state is entirely determined by T, which possesses the Markov property. Therefore, once the MDP is defined, its policy is a one-to-one mapping of each state to action, and the MDP enables learning the optimal policy corresponding to each state and the best action it should take to maximize the total expected reward R.

The optimality criterion is frequently linked through the value function $V$, which is an estimate of the value of each state, and the strategy, according to the valuation of the action in the current state $Q$ can be obtained, with $V$ representing the valuation of each state and $Q$ representing valuation of each state-action pair.

To obtain the best model policy, observe the state-action space as much as possible and use the ε-greedy algorithm to explore the actions to be executed in the present state. The agent will choose the current state with probability $p$ and will choose random actions with probability $1 - p$. Continuously interacting with the environment and adjusting its own $V$ and $Q$ functions, the agent approximates the actual $V$ and $Q$ functions. $Q$ function, so that the action predicted by the $Q$ function and selected by the model in the present state can get the most significant expected total reward.

The primary objective of the DQN algorithm is to match the $Q$ function, which reflects the greatest expected reward the environment may provide in a given condition and activity. The $Q$ function is determined by the state and activity of the system. After obtaining $Q\left( {s,a} \right)$, we may obtain the policy function. $Policy\left( s \right) = arg_{a} max\left( {Q\left( {s,a} \right)} \right)$ is a state-dependent policy function that selects the action that maximizes the value of $Q$.

**Figure 4:**
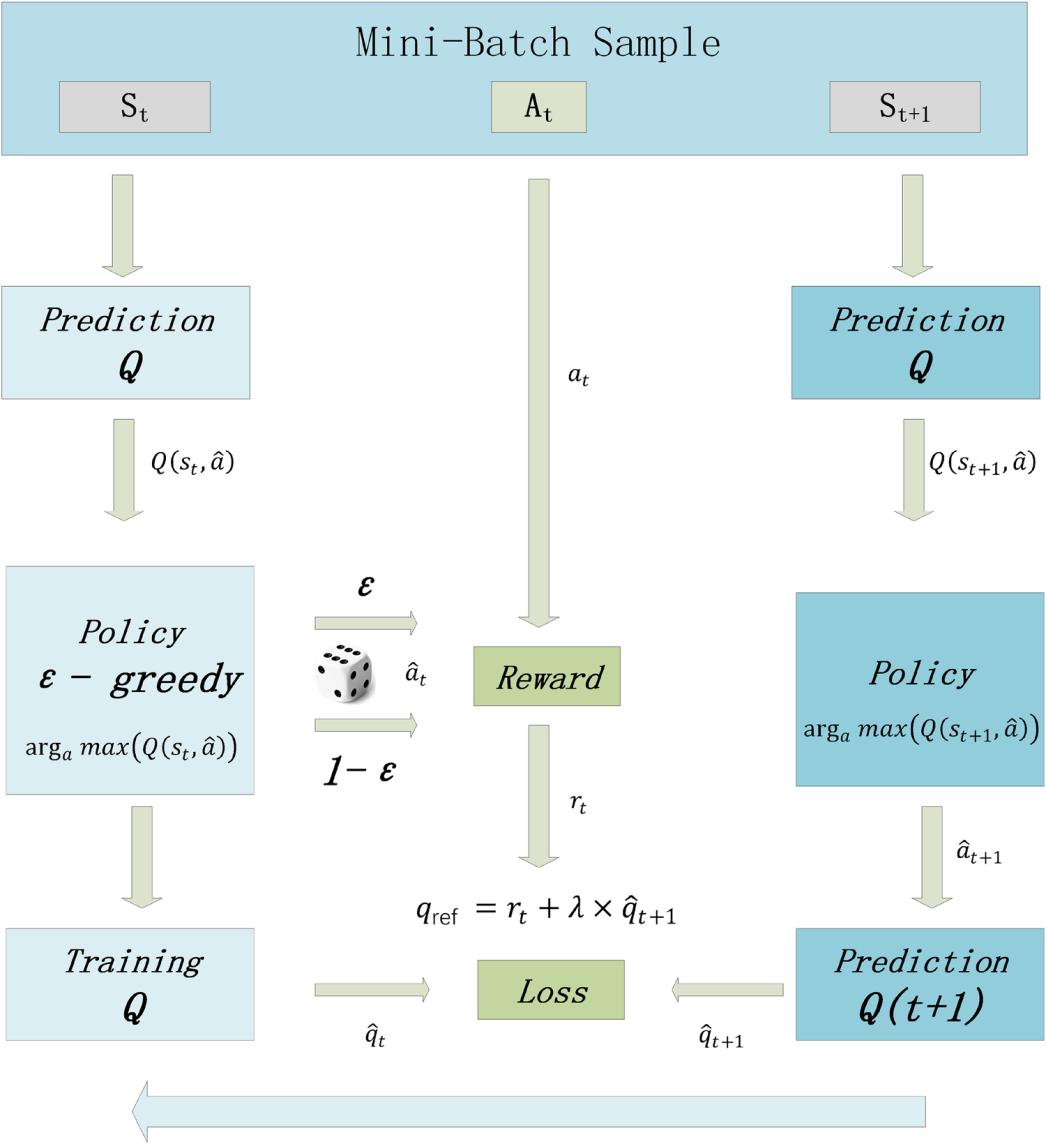
Schematic diagram of DQN model structure.

Figure 4 depicts the fundamental flow of the DQN algorithm, in which a sample of the Mini-Batch defined in the previous paragraph is fed into the model, and all Mini-Batches are regenerated at each iteration. The equivalent $Q\left( {s_{t} ,a} \right)$ and $Q\left( {s_{t + 1} ,a} \right)$ are computed based on the present respective states and $\hat{a}_{t}$. By submitting those as mentioned above, the primary fitted $Q$ function to the Policy function, the maximum $Q$ value in the current state is then determined.

$Q\left( {s_{t} ,a} \right)$ is further computed through the Policy function to obtain the maximum $Q$ value, while the next action (label) to be attempted is selected using the ε-greedy algorithm with the probability and passed into the Reward section to compare with the actual action (label) to compute the reward value, while the $Q\left( {s_{t + 1} ,a} \right)$ is also computed through the Policy function.

The $q_{t} , q_{t + 1}$ are then computed by the respective selected at and $a_{t + 1}$, and the rt calculated by $q_{t + 1}$ and reward is acquired by the reward function as $q_{ref} = r_{t} + \lambda *q_{t + 1}$, where the reward is set to a deduction factor of 0.01, which demonstrates that there is no relationship between $s_{t}$ and $s_{t + 1}$. Next, we will get $q_{t}$ and $q_{ref}$. To calculate the Loss value and back propagate through the training network in order to update the DQN model's parameters.

Algorithm 2 describes the process of RFE to select the optimal feature subset in DQN model by defining $F_{i}$ as the optimal feature subset $(F_{1} > F_{2} > F_{3} > \ldots )$, retaining the top-ranked $F_{i}$ feature subset each time, repeatedly fitting the model and evaluating the model's accuracy, and locating the Fi feature subset with the optimal accuracy to be applied to the subsequent model as the feature selection result.
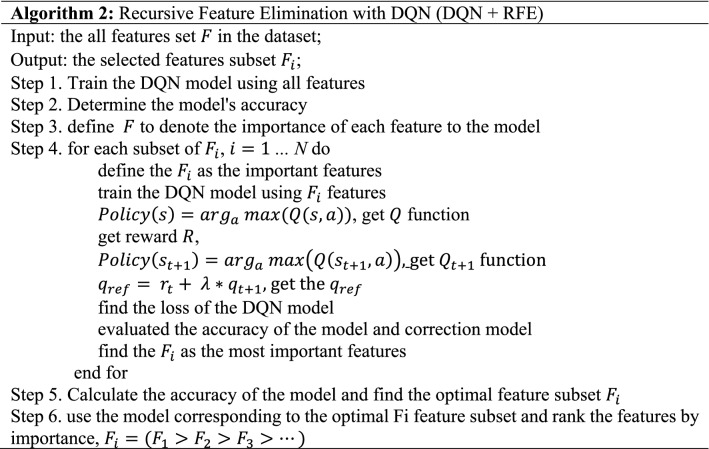


#### DRL model

**Figure 5:**
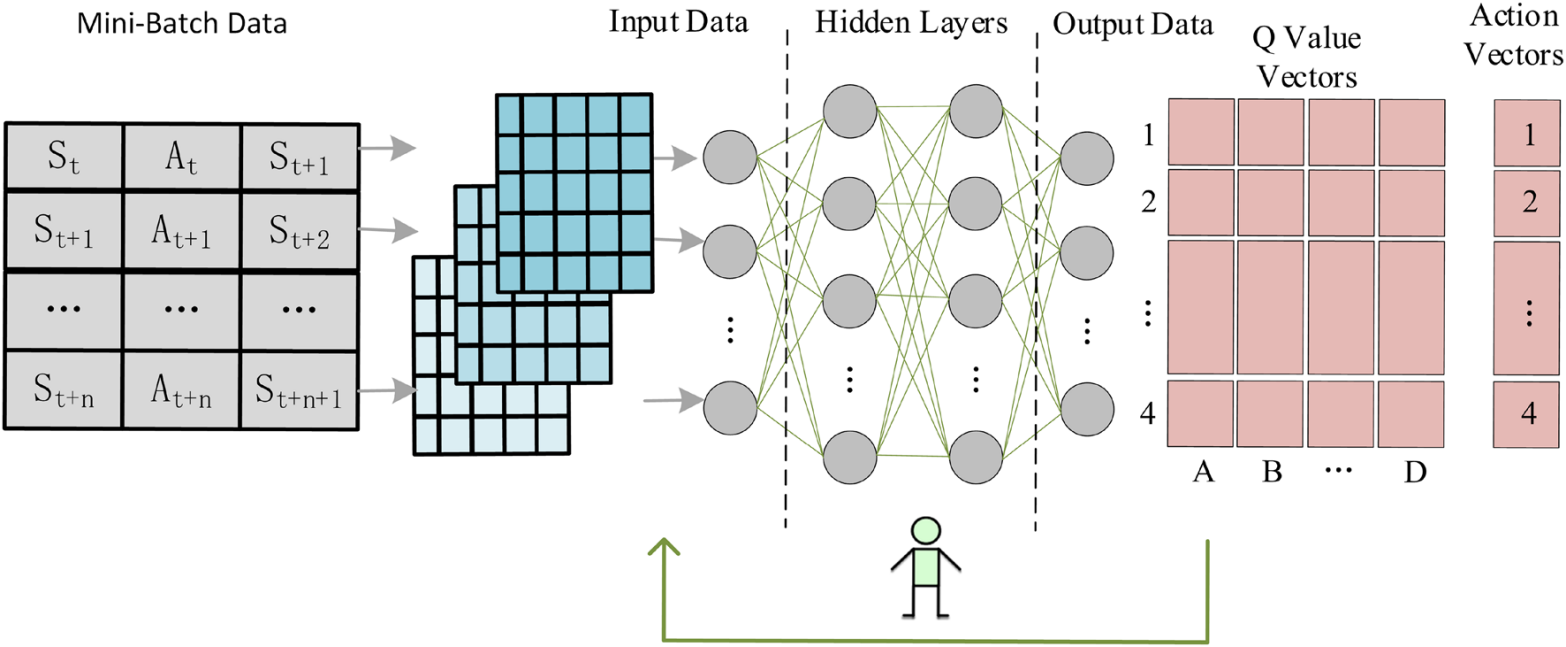
Schematic diagram of DRL model structure.

Figure 5 depicts how the DRL model was generated using the DQN method, as described in the previous section. The Mini-Batch samples selected by features are used as the model's input, and the feature values are extracted after convolutional layers. The feature values are then Flattened as the input data into the 3-layer fully connected layer, and the activation function of each layer in the fully connected network is then evaluated. Each layer’s activation function in a fully linked network is the ReLU function, which ensures that all $Q$ values calculated are positive.

The DQN algorithm is primarily applied to the fully connected layer of the DRL model, and the model will calculate the prediction $\widehat{{a}}_{t}$ and $\hat{a}_{t + 1}$ corresponding to $s_{t}$ and $s_{t + 1}$ states respectively, then the predicted action a hat t and the state st correct action at continue to compare, if they are the same then the reward is 1; otherwise it is 0, and the reward value is obtained as $r_{t}$.

Notably, the reward discount factor of the model is set to 0.01 in order to get the most excellent performance and encourage the model to focus on the present learning reward, given that the dataset is labeled and the labels are uncorrelated.

### Performance metrics

In IDS, correctly identifying attack traffic is more crucial than validating regular traffic. In addition to accuracy, one of the metrics to be considered when assessing the model's performance, we also analyze the model's performance using F1-score, precision, recall, and ROC metrics.

These metrics are produced using confusion matrices, consisting of TP, TN, FP, and FN with a grid structure that enables the visualization of the model's performance. TP stands for true positives, which indicate correctly predicted attack traffic; TN stands for true negatives, which indicate correctly predicted normal traffic; FP stands for false positives, which indicate normal traffic that is predicted as attack traffic; and FN stands for false negatives, which indicate attack traffic that is predicted as normal traffic. FN is the essential element; the lower it is, the less likely IDS is to misjudge attack traffic, and our methodology aims to minimize its value.

The basic notation of the metrics as mentioned above is described below.

*Accuracy* the number of correct predictions made by the model as a percentage of the total number of predictions.

2 $$ {\text{Accuracy }} = \frac{TP + TN}{{TP + TN + FP + FN}} $$

*Precision* this metric measures the percentage of attack traffic correctly predicted as attack traffic and is mathematically defined as follows.

3 $$ {\text{Precision }} = \frac{TP}{{TP + FP}} $$

*F1-Scores* This metric is a combined form of model accuracy and sensitivity, and is a reconciled average of model accuracy and sensitivity. In an unbalanced dataset, better F1-Scores indicate fewer misclassified flows, and this metric is the focus of our study.

4 $$ F1{ } = \frac{TP}{{TP + \frac{1}{2}\left( {FP + FN} \right)}} $$

Receiving operating characteristics curve (ROC): ROC is a combination of response sensitivity and continuous specificity variables that may indicate the link between sensitivity and specificity; the greater the area of the curve, the better the model’s performance.

## Results

This section will first provide the results of feature selection and the optimal subset of features used to extract the data while comparing the performance of the ID-RDRL model with the MLP, CNN, Logistic Regression, DDQN, and SVM ML models that have been applied to the CSE-CIC-IDS2018 dataset. The multiple models were executed on the same test set of the CSE-CIC-IDS2018 dataset without sampling the training data or modifying the original dataset, indicating the generalization of the IDS in terms of its capacity to recognize novel network traffic.

F1-score, accuracy, precision, and recall are the measures used to assess the performance of IDS. Since the CSE-CIC-IDS2018 dataset is uneven in terms of the number of samples from different kinds of cyber-attacks, we focus more on the performance of the F1-score, which is more suited to unbalanced datasets. Moreover, to demonstrate the performance of ID-RDRL in recognizing network traffic attacks, we identify the seven kinds of data traffic in the dataset depending on whether the network traffic is normal traffic or attack traffic (Binary).

### Feature selection results

**Figure 6:**
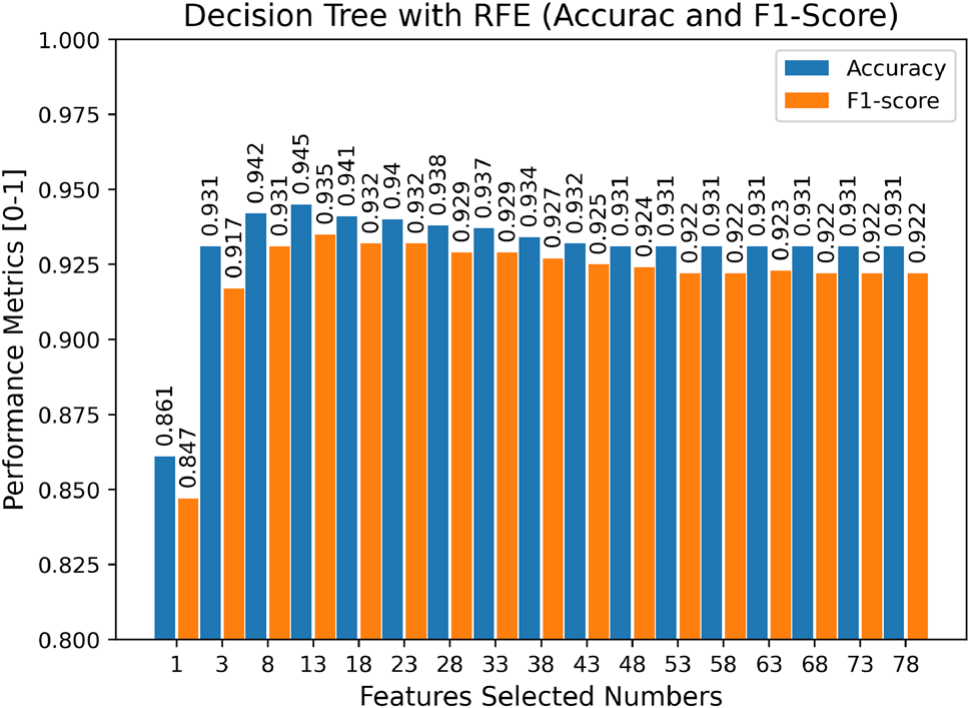
The results of Decision Tree with RFE (Accuracy and F1-Score).

We select the feature in the dataset using the DT + RFE model, where the number of RFE features picked ranges from 1 to 78, the display is spaced by five features, and the ideal number of features and feature subset is determined based on the F1-score and accuracy, as seen in Fig. 6. RFE ranks the specified characteristics based on their significance in assessing whether the traffic is attack traffic. The features picked by RFE are ordered according to their relevance in identifying whether the data is attack traffic, and the best number of feature subsets is determined to be 13 based on Fig. 6 and Table 3, respectively.

**Table 3:** Top-ranked selected features in the dataset.

SN	Features	SN	Features
1	Dst Port	8	Fwd Act Data Pkts
2	Init Fwd Win Byts	9	Bwd IAT Mean
3	Fwd Seg Size Min	10	Bwd IAT Std
4	Bwd IAT Tot	11	Bwd IAT Max
5	Fwd Pkts/s	12	Bwd IAT Min
6	Bwd Pkts/s	13	Pkt Len Std
7	Bwd Seg Size Avg	–	–

For the CSE-CIC-IDS2018 dataset, we have a problem with multiple classifications. The results of multiclassification can be presented in two ways: aggregated or one versus the rest.

In the instance of one vs. the rest, each individual class (label) is compared to all other classes, resulting in a sequence of binary classifications (one for each specific class). In aggregated instances, we return a single result that represents the average (aggregate) of all classes. Also aggregated employs several averaging techniques (micro, macro, weighted, sampling) that provide distinct outcomes. Unless otherwise specified, the performance measures (F1, accuracy, and recall) presented in this work were compiled using the weighted weighting approach, as demonstrated by Pedregosa et al.^38^.

**Figure 7:**
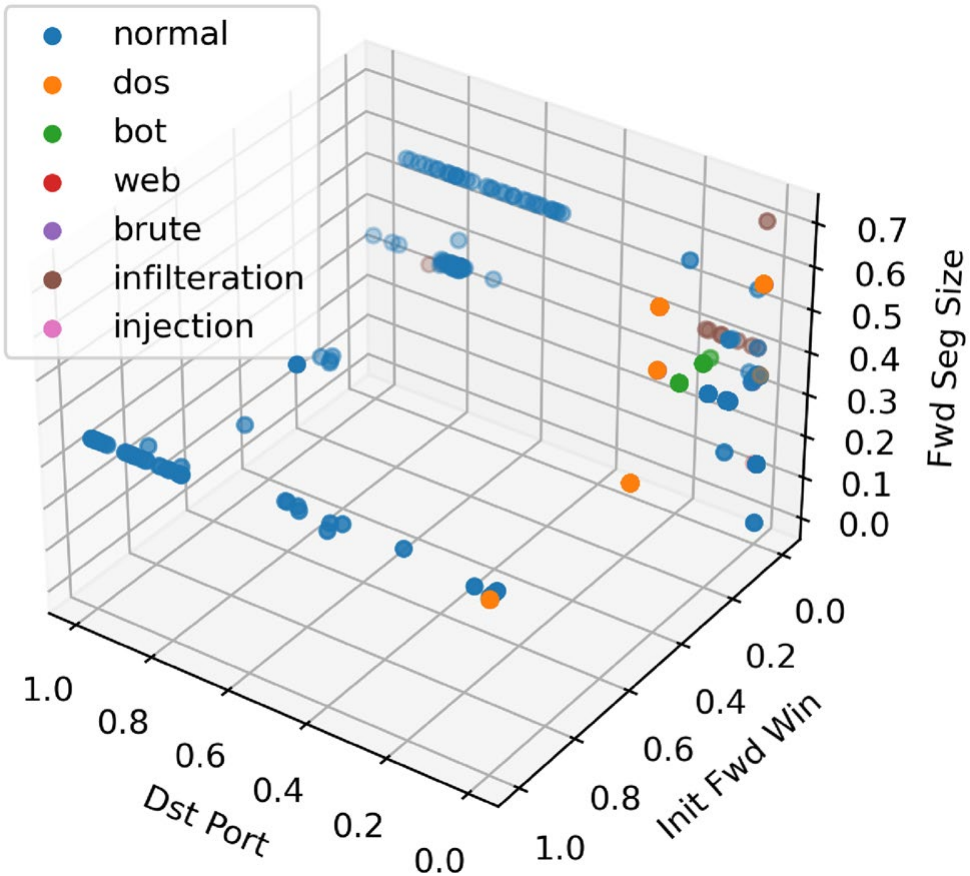
Visualization of dataset with top 3 features. Dst Port, Init Fwd Win and Fwd Seg Size features.

Due to the small number of categories, a total of 1000 data are picked, and each type of data is chosen based on the ratio in the dataset description part, as seen in Fig. 7. Although only three dimensions cannot represent the complete data, we can see the distribution of different network traffic in the potential space. We can also observe that most of the normal traffic data is on the left side of the figure, whereas most of the network attack data is on the right side of the figure, indicating that the subset of features selected by RFE can distinguish normal traffic from network attack traffic, which is the interpretability. We also discover that there is some overlap between different forms of network attacks and normal network traffic (the right side of the diagram), which might provide difficulties for our model to recognize network assaults.

**Figure 8:**
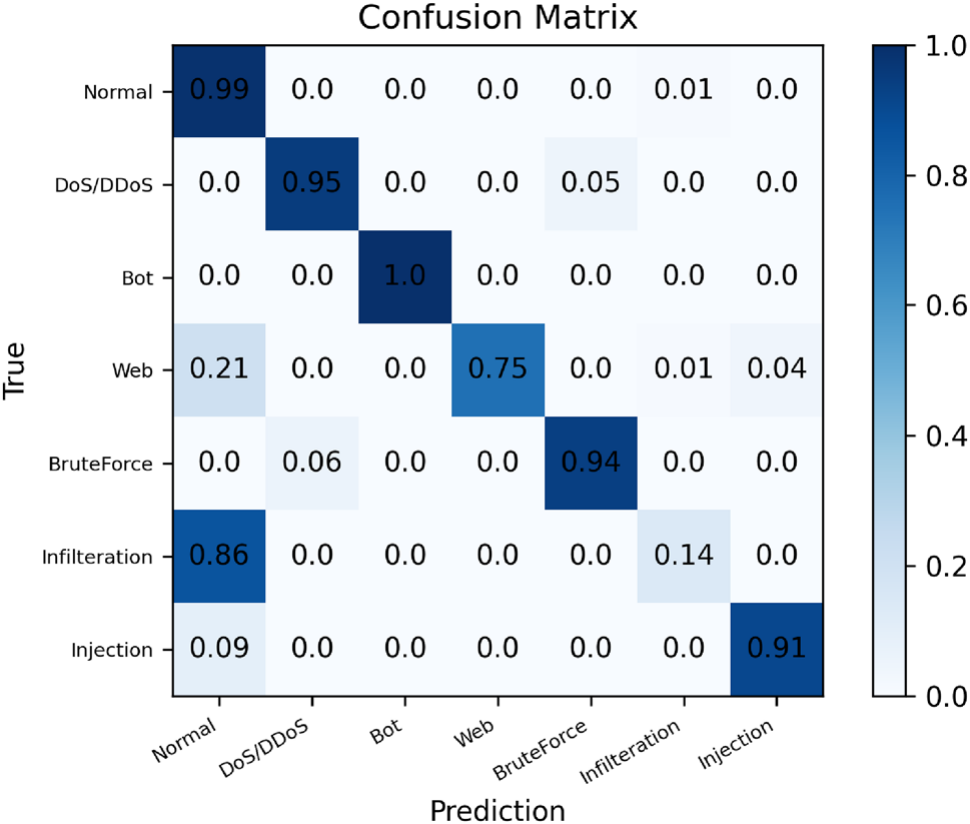
Multi-category confusion matrix (DT). The numbers in the confusion matrix indicate the proportion of samples that are classified from the original category represented by the horizontal axis to the category represented by the vertical axis.

**Figure 9:**
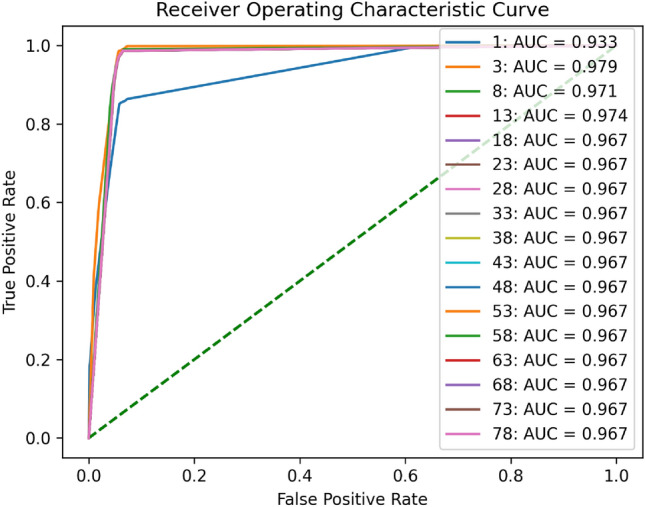
ROC of the DT with RFE. AUC is shown on the right.

Table 4 displays the Results of DT with the given characteristics, where the "13" in DT + RFE (13) refers to the optimum subset of features selected. The confusion matrix and ROC of feature selection for DT are depicted in Figs. 8 and 9, respectively.

**Table 4:** Results of DT with the selected features.

Evaluation metrics	DT	DT + RFE(13)
Accuracy	0.9312	0.9447
F1-score	0.9223	0.9354
AUC	0.9671	0.9742

We found that the performance of the DT classifier improved when the RFE-selected features were applied to the model. The overall accuracy and F1-score increased by 1.3% and 1.4%, respectively, compared to the model without feature selection. However, it did not improve in terms of AUC, which increased by only 0.6%, which may be as the RFE feature selection approach has reached its maximum level of model improvement.

Figure 8 illustrates the confusion matrix for multiple classes with 13 features. The model accurately predicts the regular, DoS/DDoS, and Bot categories, but poorly predicts the Infulteration category and mistakenly classifies them all as normal traffic. Figure 7 reveals that all network assaults in this category overlap with normal, which may contribute to the model's inability to identify this category accurately.

We used the one vs. one method to generate ROC images for two types of data, normal data and attack traffic, with different numbers of features, which helps us analyze the quality of the prediction probability. We discovered that as the number of selected features increases, the closer the ROC image is to the upper left, the larger its AUC value, and that when the number of selected features reaches 13, the AUC value remains essentially the same indicating that the prediction probability is stable. Meanwhile, we observe that the AUC achieves its maximum value when the number of features is three, contrary to our forecast that the highest AUC would be thirteen. Overall, the ROC curve demonstrates the effective detection capabilities of the DT model.

### DRL result

We altered the performance of the classifier by including reinforcement learning (RL) into the model, as explained in detail in the former part results. We achieved varying results using the subset of features filtered in the previous section as input data. As demonstrated in Table 5, the combination of the RFE feature selection approach with the application of RL increases the DT classifier’s accuracy and F1-score relative to other classifiers in the following ways: DRL + RFE achieves 96.18% accuracy and 94.89% F1-score, respectively.

**Table 5:** Results of DRL with the selected features.

Evaluation metrics	DT	DRL	DRL + RFE(13)
Accuracy	0.9312	0.9408	0.9618
F1-score	0.9223	0.9246	0.9489
AUC	0.9615	0.9746	0.9839

**Figure 10:**
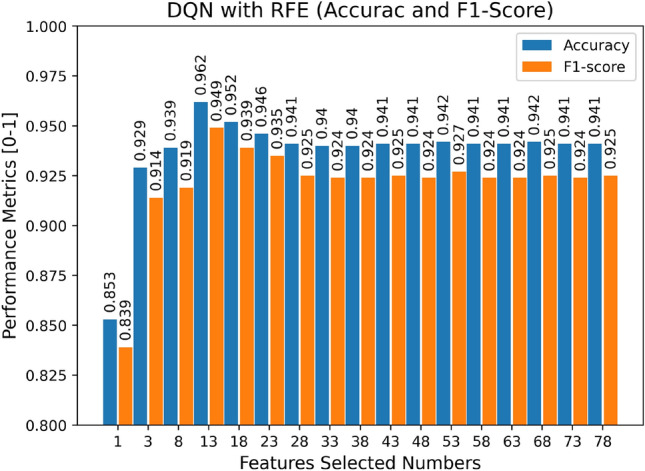
The results of the DQN Model with RFE (Accuracy and F1-Score).

**Figure 11:**
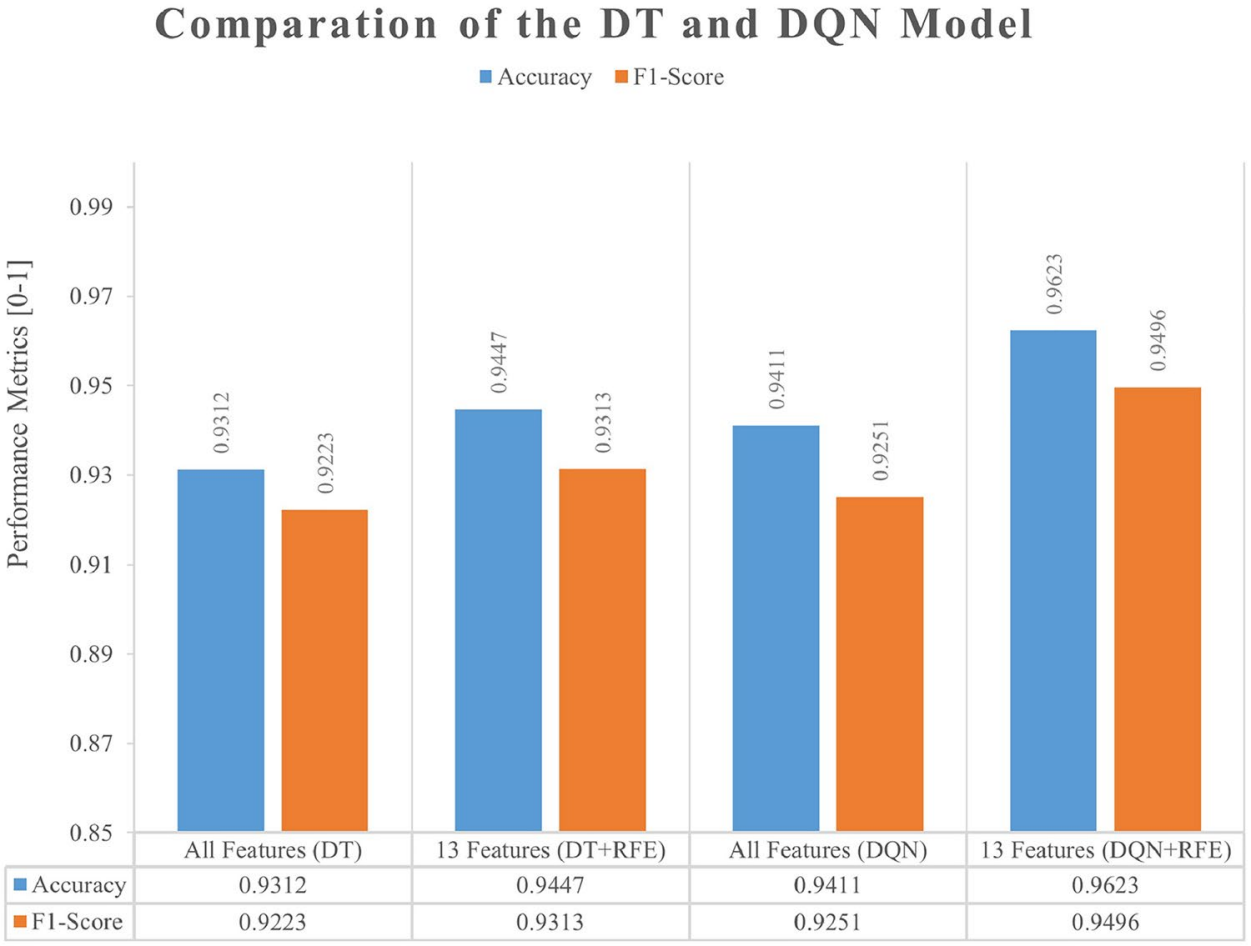
Comparison of the results of two models.

Figure 10 illustrates the impact of the number of features on the model's accuracy and F1-score after RFE ranked the significance of the characteristics for recognizing attack traffic. We can determine that the trend is comparable to the influence of the number of features on the model’s performance in the previous section, with the most excellent performance for the subset of 13 features. Additionally, we observe that the trend from 3 to 13 features is climbing and then decreasing, which differs from the trend at the exact location in the preceding section, which is likely because the enhanced DT classifier via RL can learn the previously disregarded data more precisely. Figure 11 depicts a comparison of the outcomes of the two models of DT and DQN utilizing the RFE feature selection approach. We can see that the Accuracy and F1-score of both models are enhanced when RFE feature selection is used.

**Figure 12:**
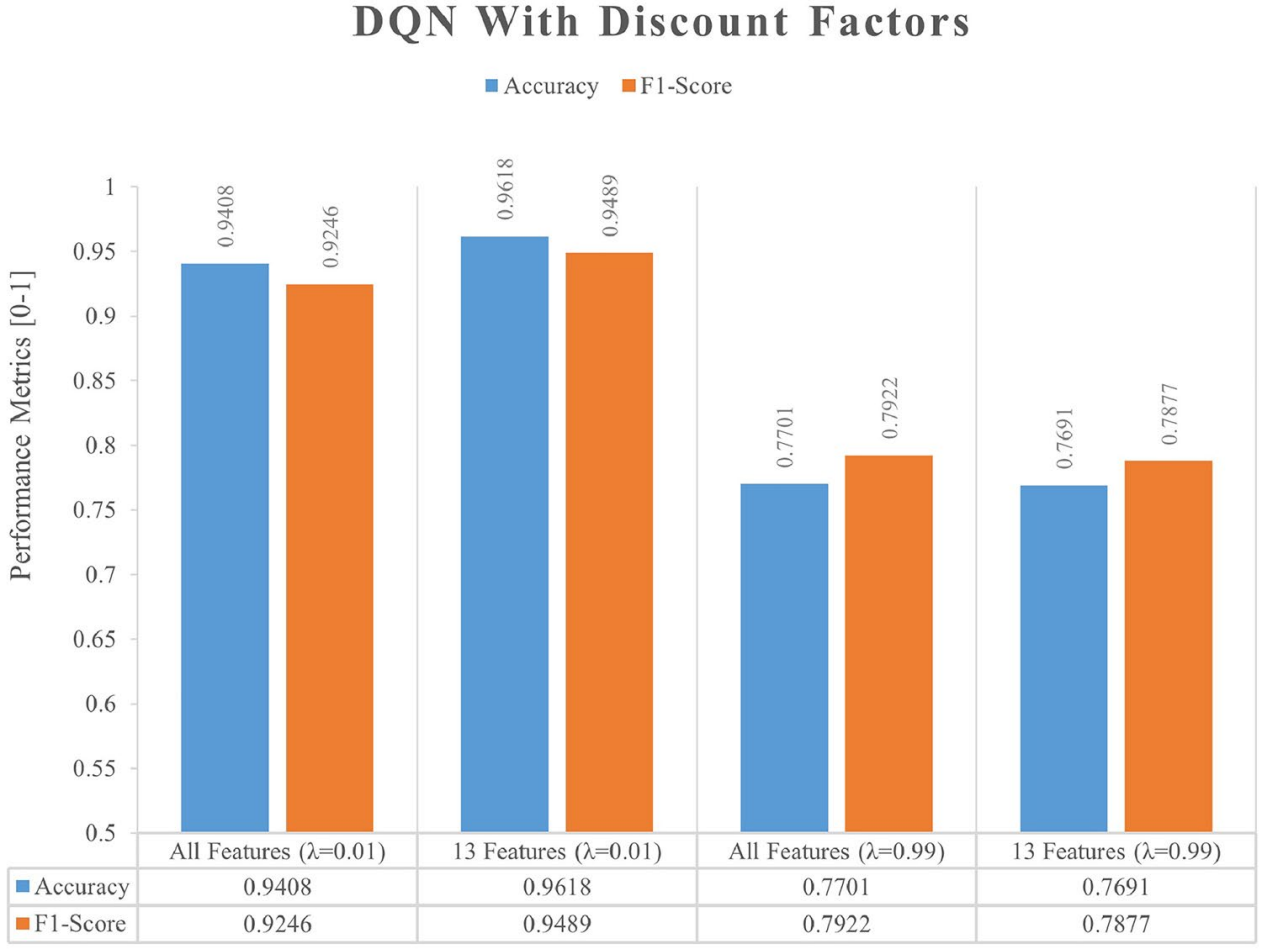
The impact of different discount factors (λ).

To further examine the impact of the discount factor in the DRL algorithm on the performance of the model, we tested the DRL model with sets to 0.01 and 0.99, respectively. As anticipated, Fig. 12 depicts the effect of employing different discount factors in the DRL model, and we achieved the most significant results with extremely low discount factors, which is mostly due to the fact that the DRL model learns relatively little from the context for the supervised learning dataset, which has low data correlation.

**Figure 13:**
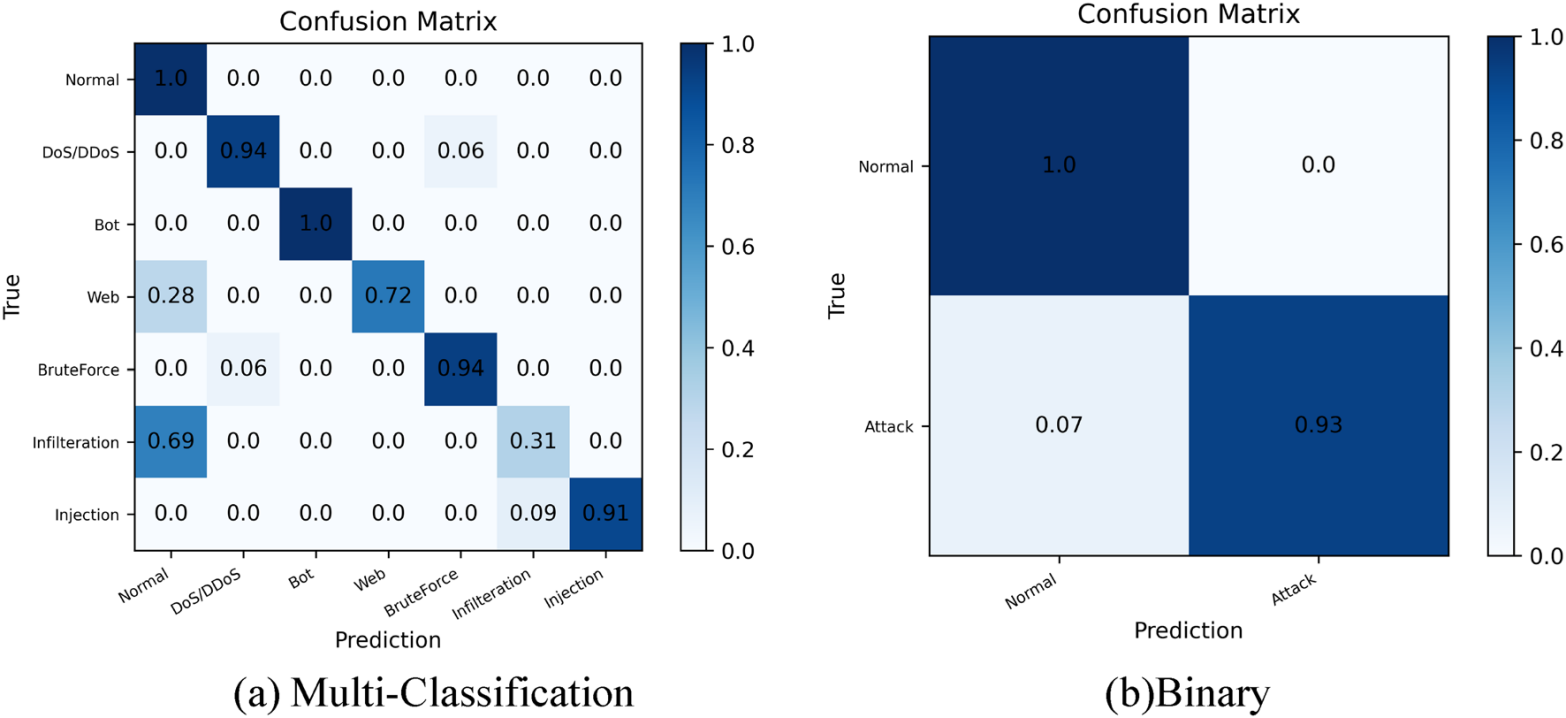
Confusion matrix diagram of the model. (**a**) Multi-category confusion matrix. (**b**) Binary confusion matrix.

Figure 13 depicts the confusion matrix plots for (a) multiclassification prediction under DQN-RFE and (b) biclassification prediction under DQN-RFE. The multiclassification is comparable to the confusion matrix plot of multiclassification prediction of DT in section DT + RFE result, and the confusion matrix plot of biclassification prediction demonstrates that all normal traffic is correctly predicted. In contrast, the probability of correctly predicting network attacks is 0.93.

**Figure 14:**
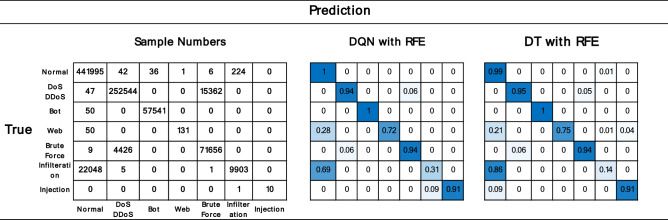
Comparison chart of the two models' multi-category confusion matrix. The left graph shows the actual number of predictions for each classification. The right plot shows the DQN and DT multi-category confusion matrix, respectively.

Figure 14 depicts comparison between the obfuscation matrix plots of the proposed DQN + RFE model and the DT + RFE model. The figure on the left depicts the actual number of obfuscation matrix plots for each category, with Normal having the most, DoS/DDoS having the second most, and Web and Injection having extremely few samples, with Injection having just 11 examples. The middle and right graphs compare the confusion matrices of the two models. The overall prediction results of DQN for many categories are comparable to those of DT, however for the Infilteration category, the prediction accuracy of our suggested model has increased from 0.14 to 0.31, indicating that our proposed model is superior.

### Comparison of different methods

In this section, the performance of the ID-RDRL model is compared with some ML models that have been applied to the CSE-CIC-IDS2018 dataset, such as MLP, CNN, Logistic Regression, DDQN and SVM ML models.

The performance of our proposed model compared to other ML methods on the CSE-CIC-IDS2018 dataset is displayed in Table 6. Our suggested DQN-RFE technique outperforms the competition, increasing accuracy by 2% and F1-score by 1.6% compared to the second-best performing approach XGBoost, where the Naive Bayes model is separated into Gaussion-NB, Bernoulli-NB, and MultinomialNB models. Their results are all dismal, whilst the other models perform rather well; XGBoost is a new ML model introduced in the past few years, and its performance is second only to our suggested model.

**Table 6:** Comparison of prediction performance and running time of multiple models.

Detection model	Accuracy	F1-score	Running time (ms)
Logistic regression	0.881	0.782	6.294
KNN	0.927	0.907	317.929
Random forest	0.836	0.735	26.870
GBM	0.934	0.921	24.717
Gaussian-NB	0.796	0.389	4.738
Bernoulli-NB	0.728	0.589	5.032
Multinomial-NB	0.558	0.498	4.496
AdaBoosts	0.946	0.906	59.217
Neural Network	0.901	0.809	30.525
XGBoost	0.947	0.933	130.918
DT	0.931	0.922	80.301
CNN-1D	0.929	0.918	98.374
DQN	0.941	0.925	110.327
DDQN	0.939	0.928	142.392
Ours	0.962	0.949	32.932

In the final column of Table 5, we compare the running time of each model and find that the performance of models with short running times is generally poor, whereas the performance of models with lengthy running times is significantly better. The Random Forest model has the longest duration at 300 ms, which is two orders of magnitude larger than the NB series model with the smallest runtime, indicating that the longer the runtime of a model, the better its performance. Our suggested model utilizes around the average of all compared models or 32.9 ms. This is mostly due to the implementation of the RFE feature selection approach, which eliminates 80% of duplicate features and drastically reduces the computation required for the model prediction process.

## Conclusion

In this paper, we propose an ID-RDRL method based on the feature selection (RFE) method and deep reinforcement learning, validate the model’s performance using the CSE-CIC-IDS2018 dataset, and compare ID-RDRL with traditional machine learning methods in terms of accuracy, F1-Score, and running time. First, RFE can choose the ideal feature subset of the original data and eliminate around 80% of the redundant features in the CSE-CIC-IDS2018 dataset meanwhile combining DT and RFE can accelerate the feature selection process; Second, the reward setting R and the learning discount factor are critical to the performance of the model in deep reinforcement learning; Third, our suggested ID-RDRL model can be useful, and our model enables IDS to work more effectively than standard machine learning approaches.

Since feature selection approaches are critical to the performance of IDS, the initial results indicate various possibilities for future research. However, how can automatically and dynamically select feature combinations be determined? Can a DRL with several bits of intelligence facilitate a more robust interaction between the cyber-attack classifier and the surrounding environment? Future research will address these issues to enhance the performance of IDSs and their applicability to datasets.

## Data Availability

The dataset investigated for this work is the public CSE-CIC-IDS2018 dataset, which can be downloaded from IDS 2018|Datasets|Research|Canadian Institute for Cybersecurity|UNB at https://www.unb.ca/cic/datasets/ids-2018.html.

## Code availability

The computer algorithms originated during the current study can be made available from the corresponding author Y.Z. on a reasonable request.
